# Development and Preliminary Evaluation of iCanPlan: A Mobile Health Application for Intimate Partner Violence Prevention in Thailand

**DOI:** 10.3390/ijerph23050670

**Published:** 2026-05-19

**Authors:** Montakarn Chuemchit, Suttharuethai Chernkwanma, Thandar Phyo, Swarnamala Kantipudi

**Affiliations:** College of Public Health Sciences, Chulalongkorn University, Bangkok 10330, Thailand; suttharuethai.c@chula.ac.th (S.C.); 6874506953@student.chula.ac.th (T.P.); swarnamala.k@chula.ac.th (S.K.)

**Keywords:** intimate partner violence, domestic violence, smartphone application, iCanPlan, Thailand

## Abstract

**Highlights:**

**Public health relevance—How does this work relate to a public health issue?**

- Intimate partner violence (IPV) remains a major public health issue in Thailand, with low help seeking due to stigma and limited awareness of services.
- This study addresses the need for accessible, confidential, and scalable interventions through a mobile health (mHealth) application.

**Public health significance—Why is this work of significance to public health?**

- The iCanPlan app demonstrates high usability, safety, and cultural appropriateness, indicating strong potential as a digital IPV prevention tool.
- It provides an innovative, evidence-based approach to bridge gaps in awareness, risk assessment, and access to support services.

**Public health implications—What are the key implications for practitioners, policy makers and/or researchers in public health?**

- Digital health tools like iCanPlan can be integrated into national IPV prevention strategies to enhance reach and support for vulnerable populations.
- Further large-scale and population-based studies are needed to evaluate effectiveness in improving help-seeking behavior and IPV-related outcomes.

**Abstract:**

Intimate partner violence (IPV) is a significant global public health issue that requires accessible, scalable, and contextually appropriate interventions. Mobile health (mHealth) technologies provide a promising platform to deliver support, information, and safety planning tools for individuals at risk of IPV. This study aimed to develop and pilot-test iCanPlan, a mobile application designed to support IPV prevention in Thailand. The application evaluates IPV risk, identifies indicators of danger, and provides a countrywide list of assistance sources. iCanPlan consists of four main components: (1) an IPV risk assessment tool, (2) a list of support resources, (3) educational materials presented in the form of infographics, and (4) encouraging quotes from well-known public figures. The app features a clean, user-friendly interface with intuitive navigation and color-coded components to enhance usability. In addition, a preliminary study was conducted with 30 experts from multidisciplinary fields, including gender-based violence research, social work, psychology, public health, and non-governmental organizations. Participants used the application for one month and subsequently evaluated it using a structured questionnaire based on heuristic evaluation principles. The questionnaire assessed usability, safety features, content quality, cultural appropriateness, language clarity, ethical considerations, and overall evaluation using a five-point Likert scale. Data was analyzed using descriptive statistics (mean and standard deviation) in SPSS. The findings demonstrated excellent performance across all domains, with high mean scores for usability (M = 4.93), safety features (M = 4.73), and content quality (M = 4.82), while cultural appropriateness, language clarity, ethical considerations, and overall evaluation achieved perfect scores (M = 5.00). These results indicate strong agreement among experts regarding the application’s usability, safety, and relevance. The study highlights the potential of iCanPlan as a culturally appropriate and user-friendly digital intervention for IPV prevention. Further research involving the target population is needed to evaluate its effectiveness and long-term impact on help-seeking behavior and IPV-related outcomes.

## 1. Introduction

Intimate Partner Violence (IPV) is a key international problem that can be identified as a major public health, gender equity, and human rights concern [1]. The global estimates of the World Health Organization indicate that close to one out of every three women all over the world had experienced physical and/or sexual violence by an intimate partner at some point in their life [2]. This type of violence results in significant physical, psychological and socioeconomic effects, such as injury, chronic pain, depression, anxiety, post-traumatic stress disorder and decreased productivity [3,4,5]. IPV also has an influential impact on family and child wellbeing, and it is a continuing cycle of violence [6,7]. Despite recent advances in policymaking and advocacy procedures, the scale and prevalence of IPV point to the urgency for preventive and supportive actions that can be implemented on a grand scale and in a way that will be responsive to the cultural context [8,9].

IPV is still a serious problem in Southeast Asia, and the level of this threat is influenced by social constructs, family forms, and gender expectations [10,11]. About 27% of all ever-partnered women (aged 15–49) in the world have suffered physical and/or sexual IPV during their lifetime, and 13% in the past 12 months [12]. There have been similar concerns reported in Thailand where research shows that substantial numbers of women have experienced lifetime and past-year IPV [13]. In Thailand, one out of every six women has reported lifetime IPV, which is most likely to be an underestimate because of under-disclosure and stigma [14,15]. Studies carried out in both urban and rural areas indicate that various women consider partner violence as a secret or household affair, usually dictated by cultural beliefs that strongly encourage women to keep the family intact, to be tolerant, or conceal marital dark secrets [15,16,17]. These norms may discourage survivors from acknowledging abusive behavior, accepting that they are at risk or seeking outside help [13,17].

The dynamics that affect help-seeking behavior among the survivors remain complex, as revealed by recent studies [13,15]. In a qualitative study published in 2024, the authors concluded that Thai women who had experienced IPV in the last six months infrequently accessed formal assistance system (i.e., health professionals, police, legal) services [16,18]. Several barriers to help seeking were identified, including fear of social judgment, the desire to preserve family unity, financial dependence on partners, and concerns regarding child custody and family disruption. Many survivors also reported uncertainty about where to seek help, whom to contact, and what services were available, which further delayed access to timely support and assistance [16].

Similar trends have been observed globally. For instance, a study was carried out among individuals who survived IPV in India in 2024 and found that only around 14% of the women who suffered physical or sexual IPV sought any type of assistance. For those who asked for support, informal support from family members or friends was much more prevalent than formal services [19,20]. Similarly, a 2025 global study across low- and middle-income countries revealed socioeconomic, educational, ethnic and childhood violence exposures were associated with help-seeking differences [21]. Collectively, these findings suggest that structural, social, and cultural barriers play an important role in influencing survivors’ help-seeking behaviors and access to formal support services [21,22].

The barriers to seeking help are not only limited to cultural and emotional factors but also include practical challenges. Many survivors lack awareness of available support services, are uncertain about how and where to access assistance, and may not know which organizations provide confidential and nonjudgmental support [23,24]. These restrictions are even greater in rural or marginalized communities where working women live [24]. In addition, fear of retaliation, economic disengagement and fears related to social stigma also discourage the survivors from taking actions towards safety. This has led to the fact that many cases go undocumented, with survivors still under threat and not able to seek vital support systems [25,26,27].

Although there are several governmental and non-governmental based IPV support services available in Thailand, such services are unevenly distributed and not continuous across the regions [28]. Many survivors may not know what support is available, may have trouble identifying support services, or may have issues accessing services due to confidentiality, transportation, financial dependence, or fear of stigma [29]. Access issues might be more severe in rural and less-serviced areas where there is limited access to specialized IPV service. In addition, there is a lack of integrated digital tools that are available in Thai, which include IPV self-assessment, education and safety planning, and referral services in one easy-to-access platform [30]. These gaps reveal the need for culturally tuned and readily available digital interventions that can assist IPV survivors in Thailand.

Mobile technology has been an exciting initiative that has appeared in the last few years that can assist in overcoming the obstacles to IPV assistance [31]. As more people in Thailand use smartphones, mobile and digital platforms provide a somewhat discreet, convenient, and possibly empowering means to have survivors access information and support without explicit exposure or reproach [32]. Research on mobile- and web-based interventions to prevent violence and support survivors indicate that the mentioned resources might be effective in terms of raising awareness, helping the affected individuals self-evaluate, and offering confidential access to services [30,33]. Initial signs indicate that digital tools can also aid in alleviating isolation, positively affect mental health outcomes, and enhance the intention to seek help, especially in women who may not initially feel safe or comfortable approaching formal support systems [33,34]. Previous studies examining digital safety planning interventions like myPlan have shown effectiveness in outcomes such as better safety decision making, decreased decisional conflict, and increased help seeking among women who experience IPV [35].

While a few digital intervention tools have been culturally tailored and developed in Thailand, few mobile applications are specifically designed and culturally adapted to the Thai context and have been shown to have promising results in previous digital interventions on IPV [35,36]. Unlike current IPV-related apps, iCanPlan combines IPV self-assessment, culturally relevant educational information, directories of support services in the area, and safety information all in one Thai-language application. At the same time, the application was crafted to address the barriers that commonly face survivors in Thailand, such as the lacking awareness of available services, fear of losing confidentiality, and problems with access to formal support systems. These features make iCanPlan a context-specific digital intervention, focused on the needs of Thai IPV survivors.

It is against this environment that the iCanPlan development serves as a response to an evident and urgent need. The purpose of the application is to offer a safe, convenient, and easily accessible application that would assist people to be aware of the indicators of intimate partner violence, assess their own cases, and find the right support services in Thailand. iCanPlan aimed to empower survivors with knowledge and confidence through this process by incorporating IPV evaluation instruments, educational resources and a comprehensive national directory of support groups throughout the country. The application emphasizes clarity, simplicity, and discretion in its design to encourage use among individuals who may be fearful, hesitant, or unaware of available support services, including healthcare services, legal assistance, police protection, counseling services, emergency shelters, and child and family support resources in Thailand.

The current paper outlines the process, design and execution of iCanPlan. It describes the theoretical framework to be followed, design needs, and technology development process, and explains the four functional areas in the application and how these functional areas can be related to the existing literature on IPV and help seeking. Furthermore, the paper examines the possible implications of incorporating mobile-based tools in national IPV support systems, opportunities to enhance user engagement, and gives research recommendations for the future. The paper illustrates how free and open mobile apps can be used as sustainable and affordable assistance to IPV survivors. Moreover, the research offers pilot testing, which shows the usability, feasibility, and initial effectiveness of the application among the participants. The results of the pilot study offer empirical evidence on the acceptability of the app and the possibility to improve safety planning, decision making, and support access to resources, which supports the importance of the app in the context of future large-scale interventions.

## 2. Methodology

The current study used an applied quantitative method that included the development, implementation and pilot testing of a mobile application iCanPlan which supports individuals who are experiencing intimate partner violence in Thailand. 

### 2.1. iCanPlan Application Development and Implementation

To achieve the objectives of this study, a mobile application titled iCanPlan was developed for both Android and iOS devices. The application was designed as a practical tool to support individuals who may be experiencing intimate partner violence (IPV), by providing assessment, information, and access to resources. This section describes the development process, features of the application, and the validation of the IPV measurement tool integrated into iCanPlan.

As the information involved in IPV is sensitive, user privacy, confidentiality, and safety were paid special attention during the development of applications. The application’s user interface is designed to be discreet and user friendly, to minimize inadvertent disclosure. Confidential self-evaluation, easy exit and secure user-information handling were also features for safety. Only the research team had access to the data obtained in the study.

### 2.2. Application Features

The design of iCanPlan was guided by the principles of accessibility, user safety, and evidence-based practice. The application components were selected based on findings from the existing literature on IPV interventions, digital safety planning tools, and approaches to support survivors. Previous research has noted the critical importance of IPV screening and self-assessment tools for raising awareness and recognition of abusive behaviors, and the availability of safety planning resources and referral services has been correlated with increased help seeking and safety decision making among individuals who have experienced IPV [12,35,37]. While educational content has been identified as important for enhancing knowledge about IPV, supporting messages (both emotional and legal) may help build self-efficacy and emotional support, which can lead to greater engagement or retention in digital health interventions. The mobile application has four main elements that are aimed at delivering support to those living with intimate partner violence (IPV), covering assessment, information, resources and emotional support.

1. Self-Measurement Tool for Intimate Partner Violence

**Figure 1:**
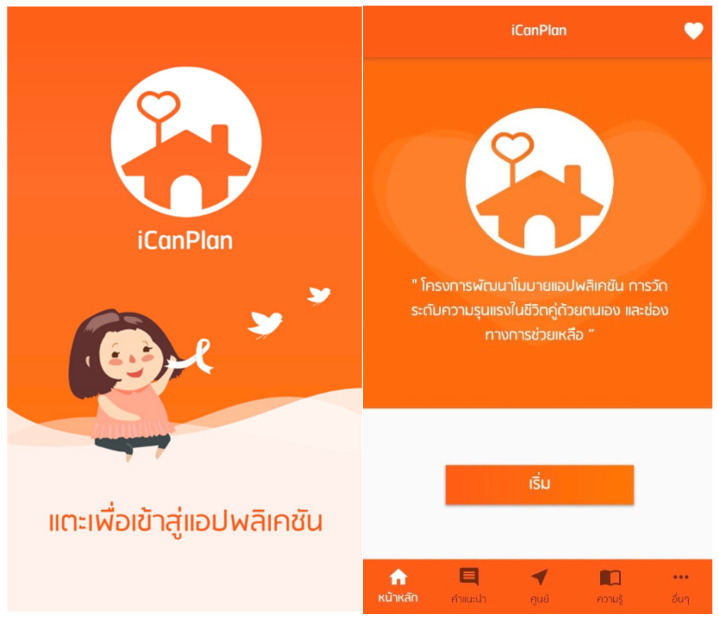
Screenshot of the iCanPlan app’s homepage activity. All Thai-language labels shown in the interface represent navigation menus and informational content in the local language (Thai) for end users. The main text translates to: “Development of a mobile application to assess the severity of intimate partner violence and promote help-seeking.”.

iCanPlan is available on smartphones and tablets and offers a confidential and user-friendly platform to the user. As shown in Figure 1, the home page of the application greets its users with a visually appealing and intuitive interface. Upon logging in, users are asked to answer two eligibility questions: Is the user 18+ years old, and has the user cohabited with a partner in the past 12 months? Eligible users then enter basic demographic details such as age, level of education, current residence and parental status.

**Figure 2:**
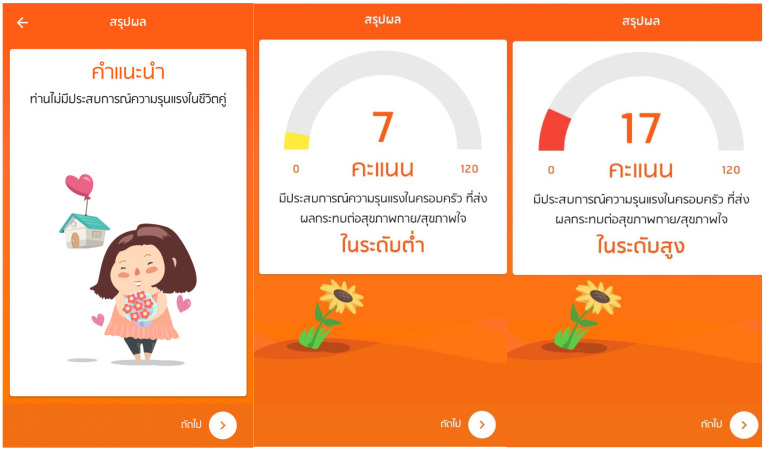
Screenshot of the iCanPlan app’s Abuse Behavior Inventory (ABI) score summary interface, which classifies users into three risk categories based on reported experiences of intimate partner violence (IPV): no risk, low risk, and high risk (from left to right). The application displays ABI scores and provides automated, personalized recommendations according to the identified level of risk. Higher scores indicate greater exposure to IPV experiences associated with adverse physical and/or mental health outcomes.

Subsequently, users go on to complete the Abuse Behavior Inventory (ABI) self-assessment tool embedded within the app. The ABI is thirty items measuring psychological, physical, and sexual violence in the past year. Once the questionnaire has been filled in, the system computes a total IPV score that classifies people into one of three levels of risk, based on their results (see Figure 2):

*No risk*: score = 0, no experience of IPV;
*Low risk*: scores between one and nine, this group represents the IPV experiences that can impact individuals from a low-level physical or mental health perspective;
*High risk*: scores of 10 or greater, that is, IPV that considerably affects physical and/or mental health.

Following the score calculation, an automatic personalized recommendation is provided by the application with the aim of conflict resolution and a healthier relational environment. Users who are classified as at risk are also provided with immediate access to appropriate support services depending on where they are located or any other area of Thailand that they choose.

2. Easy Access to Support Sources Across the Country

iCanPlan includes a wide directory of IPV support resources across Thailand so that users can seek support conveniently. Users can search for support services in an existing geographical area, or they can indicate any other province across the country. The available services fall into four categories; each color coded for ease of navigation and quick identification:

*Hotlines (24 h):* symbolized by a cantaloupe shade;
*Non-Governmental Organizations (NGOs)/Foundations*: represented by pink;
*Hospitals:* further sub-divided into community hospitals (yellow) and regional hospitals (sky blue).

**Figure 3:**
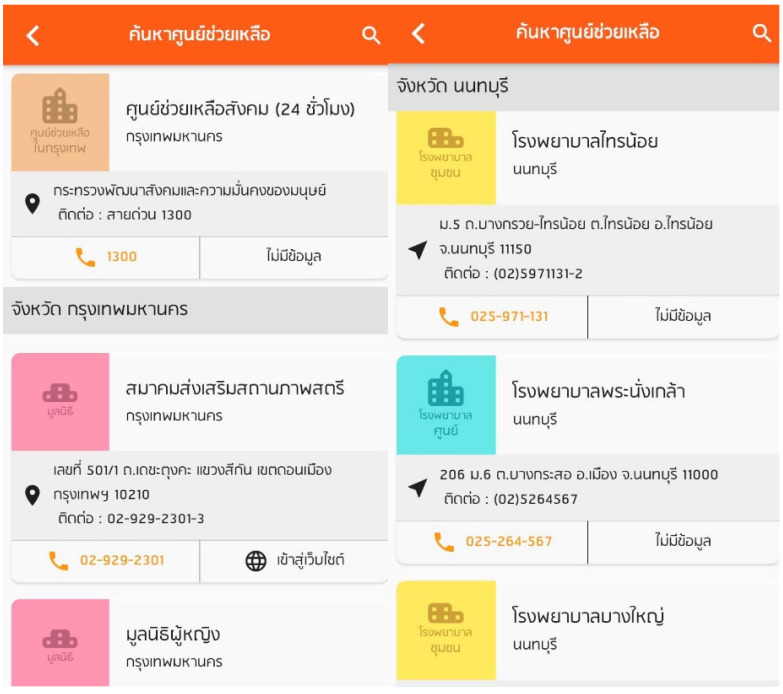
Screenshot of the iCanPlan app’s support service directory, which provides users with access to available support resources for individuals experiencing intimate partner violence (IPV). The directory categorizes services by geographical location and institution type, including social assistance centers, hospitals, women’s organizations, and emergency support services. For each support source, users can view contact information, location details, and directly access communication channels such as telephone calls and websites, facilitating timely referral and help-seeking behavior.

As illustrated in Figure 3, this color-coded system visually distinguishes the types of support that are available, making it easier for the user to search for support. The directory consists of emergency hotlines, shelters, hospitals (regional, provincial, community health centers) and non-governmental organizations specializing in IPV support.

3. Educational Resources on Intimate Partner Violence

iCanPlan offers extensive educational material about different aspects of IPV to increase user knowledge and awareness. This includes details about the various types of IPV, the risks and contributing factors of IPV, warning signs of an abusive relationship, and the short- and long-term effects of IPV on survivors and families. Furthermore, the app also provides legal information about relevant laws and protections in Thailand, information about local and global initiatives on IPV, and is aware of international observances like International Day for the Elimination of Violence Against Women and Girls. This information is easily accessible to users via a simple and user-friendly interface embedded in the app, which serves to raise awareness and help users make informed decisions.

4. Encouraging Messages from Renowned Professionals

Upon receiving their assessment results, users who are described as being either low or high risk are shown positive messages from well-known individuals in multiple professional fields, including writers, artists, musicians, academics and activists. Encouraging messages were included based upon evidence that emotionally supportive communication can increase self-efficacy, decrease feelings of isolation, and promote help-seeking behaviors for people who are experiencing violence or psychological distress. Supportive and affirming content has been found to enhance user engagement, emotional reassurance and perceived social support in previous digital and mobile health interventions, especially in vulnerable populations [36,37].

#### 2.2.1. Development and Validation of the IPV Measurement Tool

The IPV measurement tool used in iCanPlan is the Thai version of the Abuse Behavior Inventory (ABI) [38]. This instrument was derived from a broader research project titled “The Development of a Standardized Measurement Tool for Intimate Partner Violence in Thai Version” [39]. The project recruited 1441 married or cohabiting couples from four regions of Thailand, making the tool representative of various cultural and demographic contexts.

Of the total participants, 230 individuals reported experiencing psychological, physical, or sexual violence during their lifetime. These respondents were subsequently evaluated using the original ABI, which served as the reference standard for validating the Thai version.

##### Translation and Cross-Cultural Adaptation

The Thai ABI was translated with permission from the copyright holder and followed internationally accepted guidelines for the cross-cultural adaptation of self-report measures. The translation process involved forward translation by bilingual experts, followed by backward translation into the original language to verify accuracy and consistency. Any discrepancies between versions were reconciled through expert review, and cultural adjustments were made to ensure both conceptual and semantic equivalence with the original instrument. This rigorous and systematic procedure ensured that the Thai version preserved the content validity and measurement properties of the original ABI.

#### 2.2.2. Instrument Structure and Scoring

The Abuse Behavior Inventory (ABI) consists of 30 items designed to assess three primary domains of intimate partner violence: psychological, physical, and sexual violence. Participants report the frequency of each behavior experienced within the past 12 months using a 5-point Likert scale ranging from 0 (never) to 4 (very frequently). All item scores are summed to generate a total IPV score, with higher scores indicating greater severity and frequency of violent behaviors.

#### 2.2.3. Determination of Cutoff Scores

To determine an appropriate cutoff score for identifying individuals with elevated IPV experiences, a receiver operating characteristic (ROC) curve analysis was performed using the Thai ABI dataset. The analysis evaluated total ABI scores ranging from 2 to 20, and a cutoff score of 10 emerged as the optimal threshold, yielding a sensitivity of 58% and a specificity of 79%. Based on this criterion, scores of 10 or higher were classified as indicating elevated levels of IPV experiences, whereas scores below 10 indicated a lower level of IPV exposure. For practical use within the mobile application, the psychological, physical, and sexual subscales were combined into a single total score to facilitate clearer interpretation.

#### 2.2.4. Illustration of Diagnostic Accuracy

**Figure 4:**
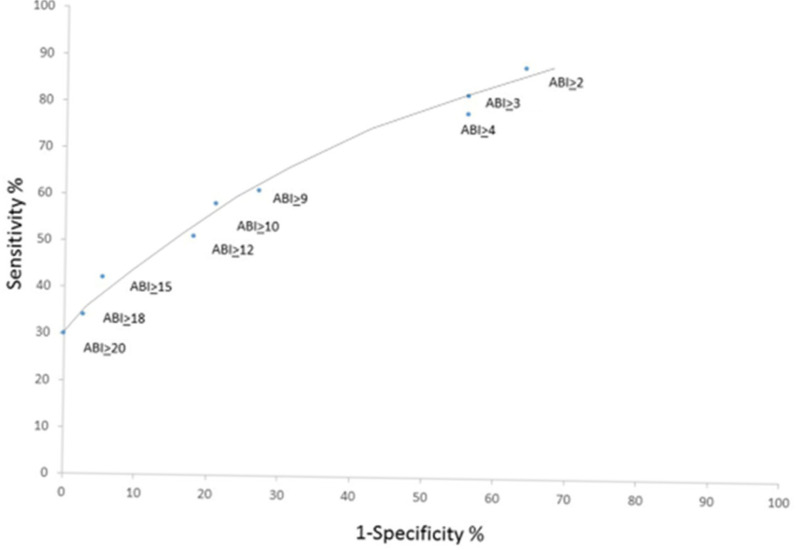
Abuse behavior inventory total score cutoffs between 2 and 20.

Figure 4 presents the receiver operating characteristic (ROC) curve for the Thai Version of the Abuse Behavior Inventory (ABI), illustrating the diagnostic performance of various total score thresholds in identifying individuals at risk of intimate partner violence. The *x*-axis represents 1-specificity (false positive rate), while the *y*-axis represents sensitivity (true positive rate). Each plotted point corresponds to a different ABI cutoff score, ranging from ≥2 to ≥20. As shown in the figure, lower cutoff scores (e.g., ABI ≥ 2, ≥3, ≥4) yield higher sensitivity but reduced specificity, meaning they identify more true cases but also produce more false positives. Conversely, higher cutoff scores (e.g., ABI ≥ 15, ≥18, ≥20) demonstrate higher specificity but substantially lower sensitivity, detecting fewer true cases. The curve shows that mid-range cutoff scores, particularly ABI ≥ 10, provide a balance between sensitivity and specificity, visually supporting its selection as the optimal threshold for classifying IPV risk.

### 2.3. Pilot Testing

#### 2.3.1. Study Participates and Recruitment

The mobile application was pilot tested using a sample of 30 participants that had prior professional experience in intimate partner violence (IPV) or gender-based violence (GBV). The participants were purposefully recruited by the researcher’s existing academic and professional networks to ensure that the application was evaluated by those that had a domain knowledge and practical field experience.

The professionals included in the eligible participants were from multidisciplinary backgrounds, such as public health, psychology, social work, gender-based violence (GBV) research, and non-governmental organizations (NGOs). The inclusion criteria for eligible participants included those with professional experience in the field of IPV/GBV prevention, intervention, research, advocacy, counseling or service delivery. The participants were chosen based on their professional skills and their awareness of issues related to IPV/GBV to ensure an informed evaluation of the usability, safety and content relevance of the application. This helped us to capture relevant feedback received during pilot testing that was informed by professional expertise.

The sampling was done through purposive sampling approach in the recruitment of participants. Invitations to participate were distributed via email and professional communication channels to individuals identified through the researcher’s academic and professional networks. Potential participants were informed about what the study was, what the mobile application was, and what they were expected to do, such as fill out the structured evaluation questionnaire.

The largest professional groups were those of NGO workers (*n* = 10), gender-based violence researchers (*n* = 10), and professionals in the field of public health (*n* = 4), social work (*n* = 3) and psychology (*n* = 3). There was a good amount of participation from both sector and researcher stakeholders on the IPV/GBV theme. Regarding the professional experience, more than 15 years of experience were reported by most participants (*n* = 15), with 11–15 years (*n* = 7) and 5–10 years of experience (*n* = 8) reported as well, indicating that the pilot testing involved highly experienced professionals (Table 1).

The application evaluation was guided by subject matter expertise, with 100% of all participants having already worked in the field of IPV or GBV. Few participants, however, had prior experience in evaluating mobile health apps (*n* = 6, 20%) while most participants did not have any previous experience in assessing mobile applications (*n* = 24, 80%). This study was voluntary. All respondents gave informed consent to participate, and confidentiality and anonymity were ensured throughout the study.

#### 2.3.2. Procedure

The pilot-testing process took place for a month. All the participants were provided with the developed mobile application and asked to install and use the application on their own mobile devices in natural environments. The participants were also advised to test the application with all its features, such as safety materials, educational materials, and usability, to have a clearer idea of how it works and how it is applicable to prevent intimate partner violence (IPV).

A structured evaluation questionnaire was used to gather the data after the one-month usage period. To make the questionnaire easy to access and answer, an online survey tool (Google Forms) was used to administer it. The survey was distributed via email, and the participants were provided with the steps to complete the evaluation.

The respondents had ample time to respond to the questionnaire, and they were reminded when required to ensure high response rate. The reason is that the responses obtained were automatically recorded and stored safely on the survey platform to be analyzed later. It is a process that allowed receiving both quantitative ratings and the opinions of experts to evaluate the usability, safety, content quality, cultural appropriateness, language clarity, and ethical considerations of the mobile application.

#### 2.3.3. Outcome Measures and Statistical Analysis

The results of pilot tests were measured based on expert review questionnaires structured on the principles of heuristic evaluation. The principles of the heuristic evaluation of digital health and mobile applications were used as the starting point of the expert evaluation questionnaire. Usability, safety features, content quality, cultural appropriateness, language clarity, ethical considerations and overall suitability of the application were evaluated. These were adopted from the literature on usability evaluation and mobile health evaluation [40,41]. The tool was created according to existing usability principles and recommendations for digital health assessment, and it included 34 close-ended questions assessed on a 5-point Likert scale ranging from strongly disagree to strongly agree. The questionnaire assessed various areas which are pertinent to mobile health application in the prevention of intimate partner violence (IPV), such as ease of use and user interface, safety, content quality and accuracy, cultural suitability, Thai language clarity and clear communication, ethics, and general assessment.

The usability domain evaluated the ease of use, navigation and uniformity of the application interface. The safety features considered privacy protection, data security, minimization of risks, and the availability of emergency support. The domain of content quality considered the accuracy, evidence base and relevance of the information to IPV prevention. The cultural appropriateness domain was used to make sure that the contents of the application were appropriate in the Thai sociocultural setting. The language clarity domain was aimed at the readability, simplicity, and non-stigmatizing use of the Thai language. The domain of ethical considerations evaluated how the application was able to reduce risks and encourage safe help-seeking behaviors. In addition, a general assessment area was used to determine the overall appropriateness of the application and its preparedness to be further tested.

The IBM SPSS software version 28.0 was used to do all the statistical analyses. The domain-specific scores were computed by taking the significance of the related items: usability (items 5–10), safety features (items 11–16), content quality (items 17–21), cultural appropriateness (items 22–24), language clarity (items 25–30), ethical considerations (items 31–32) and general evaluation (items 33–34).

The data of each domain was summarized using descriptive statistics such as mean (M) and standard deviation (SD). The increase in the mean scores depicted a higher degree of concurrence among the participants in relation to the effectiveness and suitability of the application. Cronbach’s alpha coefficients were used to measure the internal consistency of the questionnaire where a value of 0.70 or above could be seen as acceptable.

Responses from the participants were summarized using descriptive statistics. The mean and standard deviations were obtained for each item and each domain based on participants’ responses on a 5-point Likert scale ranging from 1 (strongly disagree) to 5 (strongly agree). The mean scores for the domains were calculated as the sum of all the scores of the participants for the items of each domain, divided by the number of responses. To measure the variability of the ratings of the participants, the standard deviations were calculated.

The mean scores were interpreted and presented using predetermined criteria: 4.1–5.0 was excellent, 3.1–4.0 was good, 2.1–3.0 was moderate and 1.0–2.0 was poor. These tests were a detailed assessment of the functionality of the application in the essential areas and revealed the strengths and points of improvement before additional testing on the target population.

## 3. Results

### 3.1. Domain-Wise Evaluation

The mean scores in all domains were high, which shows that the participants agree with each other on the usability and safety, as well as the overall quality of the application.

Table 2, The usability domain obtained a great score, with a total score of 888 out of 900 (M = 4.93, SD = 0.18), meaning that the participants thought the application was simple, intuitive, and easy to use. The domain of safety features was also rated highly, with a total score of 852 out of 900 (M = 4.73, SD = 0.38), which means that the application was correct in terms of privacy, security, and safety.

The content quality domain scored high (M = 4.82, SD = 0.30), which indicates that the information is accurate, relevant, and evidence based. The areas of cultural appropriateness, the clarity of the Thai language, ethical issues, and overall assessment scored the maximum 300 out of 300 (M = 5.00, SD = 0.00), meaning that all the participants agreed.

### 3.2. Item-Wise Analysis: Usability, Safety Features, and Content Quality

The usability assessment, safety-related aspects, and content quality proved to have very high scores on all items, which means that the participants agreed on the overall effectiveness and the quality of the mobile application.

Table 3, The usability and user interface domain scored very high, indicating that the application is user friendly, intuitive and well designed. Such features as ease of interface use and understandability of navigation scored perfect mean scores (M = 5.00, SD = 0.00), which means that all the participants agree on this point. Other aspects such as icon intuitiveness, layout readability, instruction clarity, and design consistency also scored high mean scores (M = 4.87–4.93) with low variability, indicating that the application is very user friendly and readable.

On the same note, the safety features domain performed well, which represents the high confidence of the application in terms of its capability to provide user safety and security. Such critical aspects as privacy protection, minimization of risks, and data security received a perfect score (M = 5.00, SD = 0.00). Other attributes, such as the presence of a quick exit option, access to emergency support, and the understandability of safety instructions, also had high mean scores (M = 4.60–4.73), but had somewhat higher variability. These results imply that although the application successfully implements the necessary safety measures, some refinements can be made to make some safety functions more accessible and understandable.

The domain of content quality and accuracy was also rated rather high continuously, which implies that the participants found the information reliable, relevant, and suitable for preventing intimate partner violence (IPV). The aspects concerning the accuracy and relevance of the content achieved perfect scores (M = 5.00, SD = 0.00), whereas such aspects as alignment with existing knowledge of IPV, clarity of language, and usefulness of information on safety planning received high mean scores (M = 4.60–4.87) with low variability. All in all, these findings indicate that the application presents reliable, comprehensible, and valuable content, and some minor areas may be improved to make the practical safety planning guidance more practical.

### 3.3. Cultural Appropriateness, Language, Ethics, and Overall Evaluation

Table 4, The areas of cultural appropriateness, language clarity, ethical considerations, and the general assessment obtained the highest mean scores (M = 5.00, SD = 0.00), which means that there was unanimity among the participants. All the items in these areas were rated as “strongly agree” consistently, which indicates that the application is highly oriented to the Thai sociocultural context, employs clear and suitable language, and has high ethical standards. The respondents further responded that the application is effective in reducing the risks on vulnerable users and encourages safe help-seeking practices. Moreover, the ideal scores in the overall assessment area indicate that the application can be viewed as entirely suitable for preventing intimate partner violence (IPV) and can be implemented with the target population.

The findings have shown that the mobile application has shown great performance in all the domains that were tested. There was a consensus especially on cultural relevance, language clarity, ethical issues, and general appropriateness.

Nevertheless, a few lower scores in certain usability and safety questions (e.g., error handling, safety advice, and accessibility of features) indicate that there are some small areas to be improved before large-scale implementation can occur.

## 4. Discussions and Recommendations

The current research was intended to design and pilot the iCanPlan mobile application for the prevention of intimate partner violence (IPV) based on an expert-based heuristic evaluation method [40,42]. The results show that the application showed high performance in all the assessed areas, which implies high acceptability, usability, and relevance to the context in the Thai culture.

The high usability scores show that the application is easy to use and navigate and is user friendly. This is especially significant in an IPV setting, where users can be stressed, afraid, or limited. The results are consistent with the recent digital health studies that focus on the importance of simplicity and easy navigation in achieving engagement and continued usage of mHealth interventions [43]. The almost perfect scores on the usability items indicate that the iCanPlan design reduces cognitive load and improves the user experience, since this is one of the most important factors to determine the success of an intervention.

On the same note, the high score of safety-related features indicates the functionality of the application in overcoming serious risks related to IPV. Such features as privacy protection, minimization of risk, and secure data processing were also unanimously supported, as they were related to the importance of guaranteeing the trust of users and their safety. The slight reduction in rating of such features as emergency access and safety guidance implies what features can be improved to make the product more user friendly. These results are in line with the recommendations by the World Health Organization, which states that digital IPV intervention should focus on confidentiality, discrete access, and user safety [2,44].

Ethical issues and safety issues have been considered when developing the IPV-related information and pilot testing it. The purpose of the study, the voluntary participation, the confidentiality assurances and the right to withdraw from the study prior to informed consent were explained to the participants. The application was designed with a user-friendly and safe interface, with the features being user friendly and the buttons clearly visible and easily accessible; the “closed” button is easily reachable for quick closing, and the emergency support button is easily visible and reachable, all with the aim of reducing possible risks to the users. User privacy protection, as well as minimizing the potential for unintended disclosure, has also been considered in the context of digital IPV interventions with vulnerable populations.

The findings on the quality of content further illustrate that the application is free of errors and offers evidence-based and relevant information, which is fundamental in preventing and creating awareness of IPV. Abuse Behavior Inventory (ABI) and other tools that have been proven to be effective, plus learning and assistance resources, further enhance the validity of the intervention. This is in line with the recent research that evidence-based digital tools enhance user trust and intervention effectiveness [45].

A perfect agreement in the appropriateness of the culture and clarity of the language is one of the most remarkable results of this research as it means that the application is properly adapted to the Thai sociocultural environment. Intervention tailoring to culture is a key determinant of IPV interventions’ success since it determines acceptability, relevance, and involvement. This has been reflected in recent studies that have indicated that mHealth interventions adapted to various cultures are more effective in dealing with gender-based violence in different populations [46,47].

The expert-based heuristic evaluation was a structured and effective methodological approach to evaluate the application before implementing it on a large scale. Although this method produces quality feedback, it is mostly based on the views of experts and not the experiences of the end users. However, it is commonly advised that expert assessment should be the first stage in the development of digital health interventions to detect usability and safety problems at the early stage [48,49].

Continuous institutional support, technical maintenance, periodic updating of content and intersectoral collaboration will be needed for long-term sustainability. Referral processes and continuity of care may be enhanced for IPV survivors if they can be integrated with healthcare systems, social welfare agencies, legal support and community-based organizations. Multilingual adaptations, extension of the service databases, user training and sustainable funding mechanisms might also be necessary for the application to be scalable to allow it to be implemented in various parts of Thailand.

Overall, the study is relevant as it adds to the expanding body of technology-based IPV prevention, especially in the low- and middle-income populations, where the accessibility to support services can be low. The results indicate that a free and open-source mobile application may be a convenient, scalable, and user-focused IPV prevention support tool.

### 4.1. Recommendations

Resting on the results of the research conducted, it is possible to suggest several recommendations regarding practice, implementation, and future research. To begin with, the application should be improved in terms of safety-related features. In particular, the emergency access functionality should be enhanced, and the safety guidance should be made more transparent to make it usable in the critical scenarios. Offline functionality should also be considered as a way of making it accessible in low-connectivity environments, which is especially applicable to users in resource-limited environments. Moreover, the application can be enhanced with interactive features, e.g., reminders and personalized safety planning tools, which can help users stay engaged and use the application regularly. Updates that are regular should be maintained to make sure that the available resources and legal information are accurate and relevant.

Implementation- and policy-wise, the application can be incorporated into national intimate partner violence (IPV) prevention programs and medical systems. It is necessary to collaborate with non-governmental organizations (NGOs), healthcare providers, and government agencies to have a wider spread and create awareness about the application. Moreover, one should make sure that the application is adjusted to international standards, especially those suggested by the World Health Organization regarding digital health interventions and violence prevention.

To conduct a study that will examine the usability and efficacy of this tool regarding the actual population, especially victims of IPV, it is suggested to implement large-scale studies in the future. Behavioral and psychosocial outcomes (help-seeking behavior, safety planning practices, and mental health improvements) also need to be evaluated. A mixed methods approach that involves quantitative and qualitative data would have given a better picture of user experiences. In addition, longitudinal research is required to examine the long-term use of the application and its long-term effects on IPV-related results.

### 4.2. Strengths and Limitations

The present research has a few strengths. It incorporated a multidisciplinary team of professionals who had a long history of experience in intimate partner violence and gender-based violence, facilitating an informed evaluation with the help of the corresponding professional experience. Another feature of the study was a structured and theory-grounded evaluation framework which gave the opportunity to make a systematic evaluation of numerous areas, such as usability, safety, content quality, cultural appropriateness, language clarity, and ethical considerations. Moreover, the incorporation of a validated instrument of IPV measurement, the Abuse Behavior Inventory (ABI), increases the scientific accuracy and validity of the application.

Nevertheless, there are a few limitations that must be considered. The findings do not have a large sample size (*n* = 30), which decreases its generalization. Also, the use of expert assessment and the absence of the target population limit the opportunity to evaluate real-life usability and user experiences. The pilot study is cross-sectional, and thus it cannot be used to assess long-term involvement or effectiveness. Another limitation relates to the presence of very high mean scores across several evaluation domains, including some domains with perfect agreement among participants. These results could reflect good interrater reliability for the application’s relevance and usability, but response bias and ceiling effects should be noted. Responses to the evaluation may also have been socially desirable, or tendencies toward rating positively by the evaluators may have affected the rating. Larger and more diverse groups of end users from the target population should be included in future studies for more diverse and external validation of the application. Lastly, the research has not evaluated the actual behavioral outcomes, including changes in help-seeking behavior or decreases in IPV risk, which should be considered in future studies.

## 5. Conclusions

The results of this pilot study prove that the iCanPlan mobile application is a very useful, safe, and culturally relevant tool in helping to prevent IPV. The ever-good rating in all spheres means that the application is properly designed and corresponds to the latest best practices in the field of digital health interventions.

Notably, the research also recommends the possibility of mobile health technologies to offer affordable, confidential and scalable services to victims of intimate partner violence. Although there is a need to make certain enhancements to certain usability and safety aspects, the overall outcomes indicate that the application can be further tested and introduced to the target population.

To sum up, iCanPlan is a promising digital innovation which can help to enhance the work of IPV prevention, especially in a resource-limited environment, and provide a resource-based support platform that is user-centered and evidence-based.

## Figures and Tables

**Table 1:** Expert characteristics (*n* = 30).

Variable	Category	*n*	%
Professional Field	NGO Worker	10	33.3
	Gender-based Violence Research	10	33.3
	Social Work	3	10.0
	Public Health	4	13.4
	Psychology	3	10.0
Years of Experience	5–10 years	8	26.7
	11–15 years	7	23.3
	>15 years	15	50.0
Experience in IPV/GBV	Yes	30	100
	No	0	0
Previously Evaluated Mobile Health Apps	Yes	6	20.0
	No	24	80.0

**Table 2:** Domain-wise evaluation of the mobile application (*n* = 30).

Domain	Total Score	Mean (M)	SD	Interpretation
Usability	888	4.93	0.18	Excellent
Safety Features	852	4.73	0.38	Excellent
Content Quality	723	4.82	0.30	Excellent
Cultural Appropriateness	450	5.00	0.00	Excellent
Language Clarity	900	5.00	0.00	Excellent
Ethical Considerations	300	5.00	0.00	Excellent
Overall Evaluation	300	5.00	0.00	Excellent

**Table 3:** Usability, safety features, and content quality evaluation of the mobile application (*n* = 30).

Domain	Item	Total Score	Mean (M)	SD
Usability and User Interface	The app interface is simple and easy to understand	150	5.00	0.00
	The navigation structure is clear and logical	150	5.00	0.00
	Icons, buttons, and menus are intuitive and easy to use	148	4.93	0.25
	Layout, font size, and color scheme are easy to read	146	4.87	0.34
	The app provides clear instructions	146	4.87	0.34
	The design is consistent across screens	146	4.87	0.34
Safety Features	Privacy and confidentiality are protected	150	5.00	0.00
	Quick exit/hidden interface available	142	4.73	0.45
	Minimizes risk of detection	150	5.00	0.00
	Data is secure	150	5.00	0.00
	Emergency support is accessible	140	4.67	0.48
	Safety guidance is clear	138	4.60	0.50
Content Quality and Accuracy	Information is accurate and evidence-based	150	5.00	0.00
	Reflects current IPV knowledge	145	4.83	0.38
	Provides useful safety planning	138	4.60	0.50
	Language is clear and understandable	146	4.87	0.34
	Content is relevant for IPV prevention	150	5.00	0.00

**Table 4:** Cultural appropriateness, language, ethics, and overall evaluation domains (*n* = 30).

Domain	Total Score	Mean	SD
Cultural Appropriateness		5.00	0.00
The app content is appropriate for Thai cultural and social context.	150		
The language and examples used in the app are relevant to Thai women.	150		
The services and resources listed in the app are relevant to Thailand.	150		
Language Clarity		5.00	0.00
The app content is appropriate for Thai cultural and social context.	150		
The language and examples used in the app are relevant to Thai women.	150		
The services and resources listed in the app are relevant to Thailand.	150		
The app content is appropriate for Thai cultural and social context.	150		
The language and examples used in the app are relevant to Thai women.	150		
The services and resources listed in the app are relevant to Thailand.	150		
Ethical Considerations		5.00	0.00
The app minimizes ethical risks for vulnerable users.	150		
The app promotes safe and supportive help-seeking behavior.	150		
Overall Evaluation		5.00	0.00
Overall, the app is appropriate for IPV prevention.	150		
The app is ready for testing with the target population.	150		

## Data Availability

The data presented in this study are available upon reasonable request from the corresponding author. The data is not publicly available due to privacy and ethical considerations.
